# Inflammation-Induced Tryptophan Breakdown is Related With Anemia, Fatigue, and Depression in Cancer

**DOI:** 10.3389/fimmu.2020.00249

**Published:** 2020-02-21

**Authors:** Lukas Lanser, Patricia Kink, Eva Maria Egger, Wolfgang Willenbacher, Dietmar Fuchs, Guenter Weiss, Katharina Kurz

**Affiliations:** ^1^Department of Internal Medicine II, Medical University of Innsbruck, Innsbruck, Austria; ^2^Department of Internal Medicine V, Medical University of Innsbruck, Innsbruck, Austria; ^3^Oncotyrol Centre for Personalized Cancer Medicine, Medical University of Innsbruck, Innsbruck, Austria; ^4^Division of Biological Chemistry, Biocenter, Medical University of Innsbruck, Innsbruck, Austria

**Keywords:** inflammation, tryptophan, kynurenine, cancer, anemia, fatigue, depression

## Abstract

Many patients with cancer suffer from anemia, depression, and an impaired quality of life (QoL). These patients often also show decreased plasma tryptophan levels and increased kynurenine concentrations in parallel with elevated concentrations of Th1 type immune activation marker neopterin. In the course of anti-tumor immune response, the pro-inflammatory cytokine interferon gamma (IFN-γ) induces both, the enzyme indoleamine 2,3-dioxygenase (IDO) to degrade tryptophan and the enzyme GTP-cyclohydrolase I to form neopterin. High neopterin concentrations as well as an increased kynurenine to tryptophan ratio (Kyn/Trp) in the blood of cancer patients are predictive for a worse outcome. Inflammation-mediated tryptophan catabolism along the kynurenine pathway is related to fatigue and anemia as well as to depression and a decreased QoL in patients with solid tumors. In fact, enhanced tryptophan breakdown might greatly contribute to the development of anemia, fatigue, and depression in cancer patients. IDO activation and stimulation of the kynurenine pathway exert immune regulatory mechanisms, which may impair anti-tumor immune responses. In addition, tumor cells can degrade tryptophan to weaken immune responses directed against them. High IDO expression in the tumor tissue is associated with a poor prognosis of patients. The efficiency of IDO-inhibitors to inhibit cancer progression is currently tested in combination with established chemotherapies and with immune checkpoint inhibitors. Inflammation-mediated tryptophan catabolism and its possible influence on the development and persistence of anemia, fatigue, and depression in cancer patients are discussed.

## Introduction

Cancer is a leading cause of death and disability worldwide with an increasing prevalence. Patients with malignant diseases often have sustained systemic immune activation, which is linked to tumor progression and a poor clinical outcome (1, 2). Initially, immune activation is an important mechanism to prevent carcinogenesis. However, this mechanism does not seem to work properly in patients with advanced cancer. Tumor cells are able to escape immune-mediated elimination by leukocytes due to loss of antigenicity and/or immunogenicity but also by creating an immunosuppressive microenvironment and by blocking anti-tumor immune response (3). Tryptophan (Trp) metabolism appears to play an important role within the tumor microenvironment (4).

In fact, enhanced Trp breakdown, reflected by decreased Trp and elevated kynurenine (Kyn) concentrations in the peripheral blood, is often observed in cancer patients and related to tumor progression, poor clinical outcome (Table 1) and an impaired quality of life (QoL) (58, 85). Trp breakdown in patients with malignancies is primarily mediated by increased tryptophan 2,3-dioxygenase (TDO) and indoleamine 2,3-dioxygenase 1 (IDO1) activities (86). The latter is primarily activated by pro-inflammatory cytokines of the T helper 1 (Th1) type immune response, particularly interferon gamma (IFN-γ) (87). IFN-γ also stimulates the formation of reactive oxygen species (ROS) as well as the expression of GTP-cyclohydrolase I (GCH-1) in target cells. In human monocytes/macrophages, this enzyme subsequently degrades GTP to form the pteridine neopterin, which has been established as a clinically useful marker for Th1 driven immune activation (88).

**Table 1:** Altered tryptophan metabolism in different cancer types and its relations to disease severity, progression, and survival.

**Cancer type**	**Tryptophan metabolism within tumor tissues**		**Tryptophan metabolism in the peripheral blood**	
	**Findings**	**References**	**Findings**	**References**
Acute myeloid leukemia	Up-regulation of IDO1 expression upon IFN-γ stimulation was related to an impaired overall survival	Folgiero et al. (5)	Increased Kyn levels were associated with a shorter overall survival	Mabuchi et al. (6)
	Increased IDO1 mRNA expression was correlated with an impaired overall survival	Fukuno et al. (7)	Kyn/Trp ratio was increased and associated with a shorter overall survival	Corm et al. (8)
	Increased IDO1 mRNA expression was related to an impaired overall survival and relapse-free survival	Chamuleau et al. (9)		
	Increased IDO1 expression inhibited T-cell proliferation	Tang et al. (10)		
Breast cancer	High IDO1 expression was associated with TNM stage, histological grade, lymph node metastasis, progression-free survival, and overall survival	Wei et al. (11)	Trp levels predict tumor progression and were associated with overall survival	Eniu et al. (12)
	Up-regulation of IDO1, TDO2, and KMO expression was found	Heng et al. (13)	Low Trp levels and an increased Kyn/Trp ratio were found	Lyon et al. (14)
	IDO1 expression increased with higher tumor stages	Isla Larrain et al. (15)	Increased Kyn/Trp ratio was associated with higher tumor grade and elevated neopterin levels	Girgin et al. (16)
	Increased IDO1 expression promotes tumor progression and is associated with an impaired overall survival	Chen et al. (17)		
	Higher IDO1 expression was associated with an impaired overall survival in estrogen receptor positive group	Soliman et al. (18)		
	Higher IDO1 expression was predictive for a better overall survival	Jacquemier et al. (19)		
	IDO1 expression was increased and correlated with tumor stages and lymph node metastasis	Yu et al. (20)		
Colorectal cancer	Increased IDO1 expression upon IFN-γ stimulation correlated with metastasis rate and an impaired overall survival	Ferdinande et al. (21)	Kyn/Trp ratio was increased and related to high neopterin levels and lymph node metastasis	Engin et al. (22)
	Increased IDO1 expression was associated with an impaired overall survival	Gao et al. (23)	Reduced Trp levels and an increased Kyn/Trp ratio was related to high neopterin levels and an impaired QoL	Huang et al. (24)
	Increased IDO1 expression upon IFN-γ stimulation correlated with reduced T-cell infiltration, higher metastasis rate and an impaired overall survival	Brandacher et al. (25)		
Gastrointestinal tumors	Increased IDO1 expression in esophageal cancer tissues was associated with differentiation grade, TNM stage, lymph node metastasis, and an impaired overall survival	Jia et al. (26)	Trp levels were decreased and associated with elevated neopterin levels	Iwagaki et al. (27)
	High IDO1 expression was a negative prognostic factor	Laimer et al. (28)		
	Increased IDO1 expression in esophageal cancer cells was related to disease progression and an impaired overall survival	Zhang et al. (29)		
Glioma	Up-regulation of IDO1, IDO2, and KMO expression upon IFN-γ stimulation was found	Adams et al. (30)	High Kyn/Trp ratio was correlated with an impaired overall survival	Zhai et al. (31)
	Increased IDO1 expression was correlated with an impaired overall survival	Mitsuka et al. (32)	Low Trp, KYNA and QUIN levels, and a high Kyn/Trp ratio were found	Adams et al. (30)
	Downregulation of IDO1 expression was associated with a better overall survival	Wainwright et al. (33)		
Gynecological cancer	Marginal IDO expression in patients in early stage cervical cancer predicted a favorable outcome	Heeren et al. (34)	Increased Kyn/Trp ratio correlated with advanced disease, poor response to therapy, and an impaired overall survival	Gostner et al. (35)
	Increased IDO expression in endometrial carcinoma cells correlated with reduced T-cell infiltration and an impaired disease-specific survival	de Jong et al. (36)	Kyn/Trp ratio was increased and related to lymph node metastasis, FIGO stage, tumor size, parametrial invasion, and poor disease-specific survival in patients with cervical cancer	Ferns et al. (37)
	Increased IDO expression in cervical cancer cells was associated with higher tumor stage, lymph node metastasis, and an impaired overall survival	Inaba et al. (38)	Kyn/Trp ratio was increased in patients with ovarian cancer and associated with higher FIGO stages	Sperner-Unterweger et al. (39)
	High IDO1 expression in ovarian carcinoma cells correlated with reduced T-cell infiltration and an impaired overall survival	Inaba et al. (40)	Kyn/Trp ratio was increased	de Jong et al. (41)
	High IDO1 expression in endometrial cancer tissues was related to reduced T-cell infiltration, lymph node-metastasis, and poor progression-free survival	Ino et al. (42)	Increased QUIN levels and reduced KYNA levels were found in patients with primary ovarian cancer	Fotopoulou et al. (43)
	Increased IDO1 expression in ovarian cancer cells was correlated with impaired survival in patients with serous-type ovarian cancer	Okamoto et al. (44) and Takao et al. (45)	Elevated Trp levels and a decreased Kyn/Trp ratio was found and associated with elevated neopterin levels	Schroecksnadel et al. (46)
	High IDO1 expression in endometrial carcinoma cells was related to an impaired progression-free and overall survival	Ino et al. (47)		
Hepatocellular carcinoma	Increased IDO1 expression was associated with T-cell infiltration and an impaired overall survival	Li et al. (48)		
	Increased KMO expression was correlated with an impaired overall survival and an increased time to recurrence	Jin et al. (49)		
	Increased IDO1 expression upon IFN-γ stimulation correlates with metastasis rate and an impaired overall survival	Pan et al. (50)		
	Increased IDO1 expression in tumor infiltrating cells was associated with an increased progression-free survival	Ishio et al. (51)		
Kidney cancer	Up-regulation of IDO1 expression upon IFN-γ stimulation was found	Trott et al. (52)	Kyn/Trp ratio was increased and associated with a poorer progression-free survival	Lucarelli et al. (53)
	High IDO1 mRNA levels were associated with an increased overall survival	Riesenberg et al. (54) and Yuan et al. (55)		
Lung cancer	IDO1 expression was increased and correlated with TNM stage and lymph node-metastasis	Tang et al. (56)	Low Trp levels and a high Kyn/Trp ratio were associated with an increased lung cancer risk in the EPIC study; In the International Lung cancer cohort consortium (5,364 smoking-matched case- control pairs) the highest quintiles of kynurenine, Kyn/Trp, quinolinic acid and neopterin were associated with a 20–30% higher risk and tryptophan with a 15% lower risk of lung cancer	Chuang et al. (57) Huang et al. (58)
	Enhanced Kyn production and increased TDO2 expression by cancer-associated fibroblasts was found	Hsu et al. (59)	Post-induction chemotherapy increased Kyn/Trp ratio was associated with an impaired progression-free and overall survival	Creelan et al. (60)
	No associations between IDO1 expression and clinicopathological parameters were found	Karanikas et al. (61)	Low Trp levels and a high Kyn/Trp ratio were found and associated with high neopterin levels, low hemoglobin levels, fatigue, and QoL	Kurz et al. (62)
	Increased IDO1 expression by infiltrating tumor cells was related to an impaired overall survival	Astigiano et al. (63)	Low Trp levels and a high Kyn/Trp ratio were found and associated with elevated neopterin levels	Engin et al. (64)
			Low Trp levels and a higher Kyn/Trp ratio were found and related to tumor progression	Suzuki et al. (65)
Lymphoma	High IDO1 expression in tumor infiltrating immune cells was related to an increased overall survival	Nam et al. (66)	High Kyn levels and Kyn/Trp ratio were found and associated with tumor progression and a shorter overall survival in patients with adult T-cell leukemia/lymphoma	Masaki et al. (67)
	Up-regulation of IDO1 in non-Hodgkin lymphoma tissues was related to tumor progression, higher serum LDH and an impaired overall survival	Liu et al. (68)	High Kyn levels correlated with an impaired overall survival	Yoshikawa et al. (69)
	IDO1 expression was increased in stroma cells of Hodgkin lymphoma and correlated with an impaired overall survival	Choe et al. (70)	Low Trp levels and high Kyn levels were found and related to a shorter overall survival in patients with adult T-cell leukemia/lymphoma	Giusti et al. (71)
	High IDO1 expression in non-Hodgkin lymphoma tissues was related to a lower remission rates and an impaired overall survival	Ninomiya et al. (72)		
	IDO1 mRNA expression was increased in adult T-cell leukemia/lymphoma cells	Hoshi et al. (73)		
Melanoma	Increased IDO1 expression in nodal metastases was associated with an impaired overall survival	Pelak et al. (74)	Low Trp levels and a high Kyn/Trp ratio were found and associated with high neopterin levels and an impaired overall survival	Weinlich et al. (75)
	Increased IDO1 expression in nodal metastases was associated with clinical recurrence	Ryan et al. (76)	Patients who developed major depression during IFN-α therapy had a significantly higher Kyn/Trp ratio	Capuron et al. (77)
	Increased IDO1 expression in sentinel lymph nodes correlated with an impaired progression-free and overall survival	Speeckaert et al. (78)		
	Increased IDO1 expression in nodal metastases was associated with a poor survival	Brody et al. (79)		
Osteosarcoma	High IDO1 expression correlated with an impaired metastasis-free and overall survival	Urakawa et al. (80)		
Pancreatic cancer	Increased IDO1 expression upon IFN-γ stimulation correlated with lymph node metastasis and an impaired overall survival	Zhang et al. (81)	Higher HAA/HK ratio was associated with a reduced pancreatic cancer risk	Huang et al. (82)
Prostate cancer	IDO1 expression was increased and correlated with serum Kyn/Trp ratio	Feder-Mengus et al. (83)	High Kyn levels were associated with an impaired cancer-related survival	Pichler et al. (2)
Thyroid carcinoma	IDO1 expression was increased and associated with tumor aggressiveness	Moretti et al. (84)		

Higher neopterin concentrations mostly coincide with increased IDO-activation as reflected by a higher Kyn/Trp ratio (24, 46, 89, 90) and are related to tumor progression and an increased mortality rate (1, 91) in patients with malignant diseases.

Trp is essential for the growth and proliferation of all kinds of cells; therefore, local inflammation-induced Trp depletion is initially a defense mechanism of the immune system to limit growth of microbes but also of proliferating malignant cells (92). However, tumor cells seem to develop countermeasures via degradation of Trp, allowing them to escape this defense mechanism. Moreover, stimulation of IDO1 and Trp breakdown also impacts on Trp availability for immune cells over time and leads to the accumulation of Trp metabolites such as the kynurenines, which can directly modulate anti-tumor immune responses (93).

Apart from an activated immune system and enhanced Trp breakdown, patients with malignancies frequently suffer from anemia (94). Anemia is a main contributor to sustained fatigue (95), which is the most frequently reported symptom in cancer patients (96), affecting up to 78% (97). Actually, activities of daily living are mostly affected by cancer related fatigue (CRF) (98). Another common comorbidity is depression, affecting ~20% of cancer patients (99–101). All these comorbidities have been related to immune activation and the associated Trp breakdown.

This review discusses the current knowledge on and consequences of immune activation and Trp breakdown for the development and persistence of anemia, fatigue, and depression in cancer patients. Moreover, it gives an overview of possible therapeutic options for the treatment of comorbidities. At the beginning, a brief depiction of Trp metabolism and its relations to immune activation will be given.

## Tryptophan Metabolism

Trp is an essential amino acid that is required for protein biosynthesis. Therefore, it is essential for the growth and proliferation of cells. Trp must be supplied by diet or obtained from protein degradation, since it cannot be synthesized by human cells. The required daily amount for adults lies between 175 and 250 mg. Yet, the average daily intake for many individuals lies between 900 and 1,000 mg (102, 103). Thus, decreased Trp concentrations are suggested to be primarily caused by enhanced Trp breakdown.

Trp is also an important precursor for several bioactive metabolites including tryptamine, serotonin, melatonin, kynurenine (Kyn) and quinolinic acid (QUIN) and kynurenic acid (KYNA) as well as for the coenzyme NAD^+^. These metabolites are mainly generated by two different biochemical pathways.

**Figure 1:**
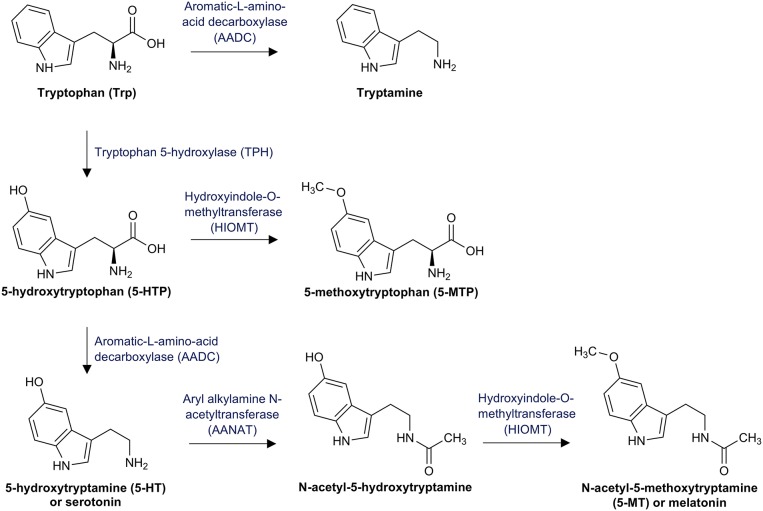
Tryptophan pathway to serotonin and melatonin: This figure illustrates tryptophan breakdown to serotonin via the intermediate product 5-hydroxytryptophan (5-HTP) and the further conversion to melatonin via the intermediate product 5-acetyl-5-hydroxytryptamine.

First, Trp can be catabolized by the enzyme tryptophan 5-hydroxylase (TPH) to 5-hydroxytryptophan (5-HTP) (Figure 1). 5-HTP is converted into 5-methoxytryptophan (5-MTP) by the hydroxyindole-O-methyltransferase (HIOMT) (104) and subsequently decarboxylated to 5-hydroxytryptamine (5-HT) by the vitamin B6 dependent aromatic-L-amino-acid decarboxylase (AADC) (105). 5-HT, better known as serotonin, is an important neurotransmitter that modulates numerous neuropsychological processes including mood, anxiety, anger, reward, and cognition (106). It is also involved in important processes outside the central nervous system (CNS), including regulatory functions in the gastrointestinal (GI) tract as well as cardiovascular and pulmonary system. Actually, over 90% of the total body serotonin is synthesized in the GI tract (107).

Although only 1% of the available Trp is converted by the Trp/5-HT pathway in healthy individuals, decreased Trp availability is associated with decreased serotonin concentrations and consequently with neuropsychologic disorders (105). In the pineal gland, aryl alkylamine N-acetyltransferase (AANAT) converts 5-HT into N-acetyl-5-hydroxytryptamine, which is further catabolyzed by the HIOMT to N-acetyl-5-methoxytryptamine (5-MT), better known as melatonin (108). Melatonin is primarily secreted at night and regulates the circadian rhythm under normal light/dark conditions (109). Finally, Trp can be directly decarboxylated by the AADC to tryptamine, which is an important neuromodulator of serotonin (110).

**Figure 2:**
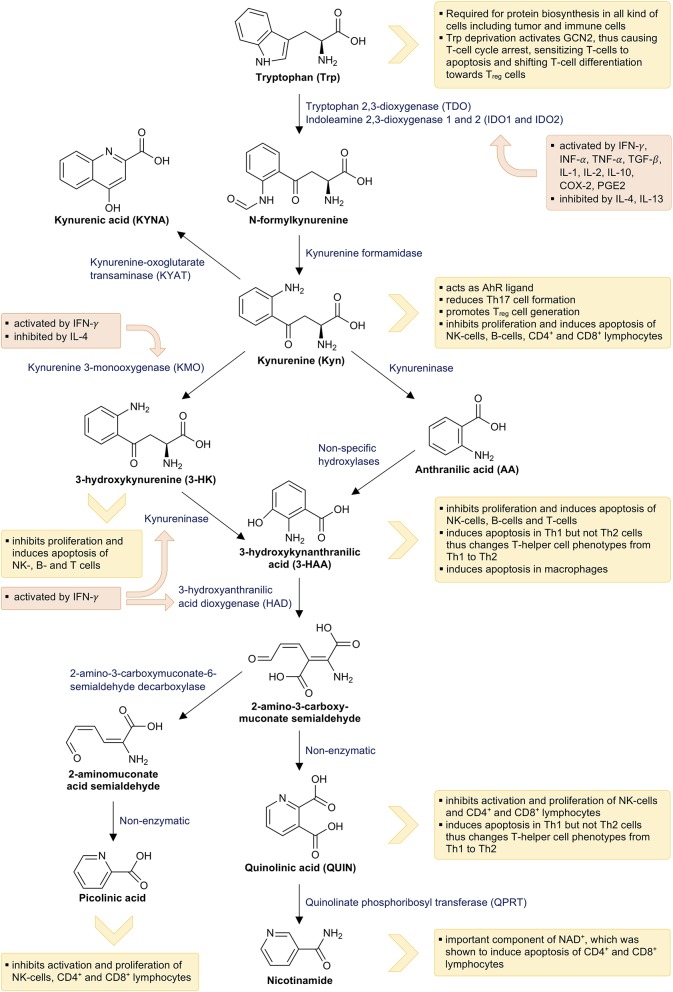
Tryptophan breakdown via the kynurenine pathway and its interactions with the immune system: This figure illustrates tryptophan breakdown via the kynurenine pathway. The orange boxes indicate the effects of immune mediators on the kynurenine pathway and the yellow boxes indicate the effects of tryptophan metabolites on the immune system.

The second and quantitatively most important pathway is the decay to Kyn (Figure 2). Approximately 90% of the available Trp is oxidized to N-formylkynurenine by either tryptophan 2,3-dioxygenase (TDO; EC 1.13.11.11), indoleamine 2,3-dioxygenase 1 (IDO1; EC 1.13.11.52), or indoleamine 2,3-dioxygenase 2 (IDO2; 1.13.11.-). N-formylkynurenine is then subsequently hydrolyzed to Kyn by kynurenine formamidase. Kyn is further catalyzed by one of the four kynurenine aminotransferases (KATs) to KYNA. It can also be hydroxylated to 3-hydroxykynurenine (3-HK) by kynurenine 3-monooxygenase (KMO) and then converted to 3-hydroxyanthralinic acid (3-HAA) by the kynureninase (KYNU). Another important enzyme of the Kyn pathway, namely 3-hydroxyanthranilic acid dioxygenase (HAD), converts 3-HAA into 2-amino-3-carboxymuconate semialdehyde, which decays non-enzymatically into QUIN. Finally, phosphoribosyl transferase (QPRT) converts QUIN into nicotinamide, which is an important component of NAD^+^ and NADP^+^ being necessary for energy production (111).

TDO, IDO1, and IDO2 are heme-containing enzymes and catalyze the first and rate-limiting step in Trp breakdown. TDO is mainly expressed in the liver and oxidizes excess Trp, thereby generating ATP and especially NAD^+^. In mammals, NAD^+^ is synthesized from Trp via the Preiss-Handler pathway in liver and kidney (112). Actually, the Trp concentration in the diet has been shown to influence the liver NAD^+^ levels (113). TDO expression is stimulated by its substrate Trp (114) as well as by heme (115) and corticosteroids (116). NAD^+^ inhibits TDO expression, thus forming a negative feedback loop (117). IDO1 can be expressed by many different cells, including antigen-presenting cells (APCs) like monocyte-derived macrophages, dendritic cells (DCs) and fibroblasts. Its expression is mainly induced by inflammatory stimuli such as IFN-γ, tumor necrosis factor alpha (TNF-α), IL-1, and IL-2 secreted by Th1 type cells, inflammatory cytokines of innate immune cells as well as TGF-β, IL-10, and adenosine secreted by regulatory T cells (T_reg_) (118). IDO1 expression is further stimulated by its own product Kyn via the aryl hydrocarbon receptor (AhR) (119–121) as well as by the cyclooxygenase-2 (COX-2) and prostaglandin E2 (PGE2) (122). Contrary to this, IDO1 expression is inhibited by the anti-inflammatory cytokines IL-4 and IL-13 (123, 124). Little is known about the physiological functions of the recently detected IDO2. It is primarily expressed in the liver, kidney, brain, placenta, and APCs including DCs and B cells; yet, IDO2 is significantly less active when compared to IDO1 (125). Similar to IDO1, IDO2 expression is stimulated by AhR activation (120). Interestingly, IDO2 negatively regulates IDO1 activity by competing for heme-binding (126). IFN-γ also stimulates KMO, KYNU and HAD activity (127).

## Tryptophan Breakdown via the Kynurenine Pathway Modulates Immune Response

An immunologicaly privileged milieu with a decreased reactivity to allogeneic (non-self) antigens is found in certain parts of the human body (e.g., brain, eye, testis, placenta). This immune tolerance prevents fetal rejection and immune responses against immunogenic sperms. An enhanced expression of TDO, IDO1, and IDO2, with a subsequent accumulation of Trp metabolites, is found in several parts of the human body including the placenta (128, 129), maternal and embryonic tissues in early conceptions (130, 131) as well as in the epididymis (132–134). Therefore, these enzymes are suggested to play an important role in immune tolerance. Immune tolerizing effects are also observed in the local tumor microenvironment. An enhanced Trp catabolism via Kyn pathway seems to be involved in immune paralysis against tumor cells. This may be primarily mediated by increased IDO1 expression and subsequent accumulation of Trp metabolites, since IDO1 is either expressed by many tumor cells themselves (see Table 1) or by tumor associated cells such as DCs or endothelial cells (ECs) (118).

Nearly all metabolites of the Kyn pathway affect immune activity via several mechanisms (Figure 2). Trp depletion slows down protein biosynthesis in immune cells and induces cell cycle arrest of T cells via eIF-2-alpha kinase GCN2, thus making them highly susceptible to Fas-ligand-mediated apoptosis (135, 136). Activation of GCN2 further promotes the generation of regulatory phenotypes (T_reg_) in naive CD4^+^ T cells (137). Activation of AhR by its endogenous ligand Kyn results in reduced T helper 17 (Th17) cell differentiation, while promoting the generation of T_reg_ cells (138, 139). T_reg_ cells, in turn, induce IDO1 expression in DCs, thus expanding their own population and forming a positive regulatory feedback loop (137). Th17 cells upregulate KMO expression, which reduces the availability of Kyn for AhR activation and consequently results in a reduced Th17 formation in the sense of a negative regulatory feedback loop (140). Finally, several metabolites of Trp breakdown such as Kyn, 3-HK, 3-HAA, QUIN, and picolinic acid were demonstrated to suppress the proliferation of CD4^+^ lymphocytes, CD8^+^ lymphocytes, and natural killer (NK) cells. Furthermore, they induce apoptosis of these cells probably mediated by oxygen free radicals (141–144), while 3-HAA induces apoptosis of monocytes/macrophages (145). However, apoptosis primarily occurs in Th1 cells and not in Th2 cells, thereby forming a negative feedback loop and preventing an excessive Th1 activation (141). In addition, the final product of the Kyn pathway NAD^+^ also induces apoptosis in CD4^+^ and CD8^+^ lymphocytes (146).

Apart from immune modulating properties, Kyn metabolites may also help tumors to “optimize their microenvironment”: Formation of QUIN by glioma cells was described to promote resistance to oxidative stress (147). Additionally, tumor cells might enhance their own IDO activity via an autocrine AhR-IL-6-STAT3 signaling loop (148), thereby suppressing T-cell proliferation. Upregulation of the tryptophanyl-tRNA synthetase WARS may protect Trp-degrading cancer cells from excessive intracellular Trp depletion via IFNγ and/or GCN2- signaling (149).

On the other hand, 5-MTP, which is produced by mesenchymal cells such as fibroblasts via 5-HTP, inhibits migration of cancer cells, tumor growth and cancer metastasis. This effect is probably mediated by 5-MTP derived inhibition of COX-2, which is constitutively overexpressed in cancer cells and promotes carcinogenesis (150). Therefore, reduced 5-MTP formation due to decreased Trp availability can contribute to tumor growth and cancer metastasis.

## Immune Tolerance Related to Indoleamine 2,3-Dioxygenase 1 Activation in Cancer Patients

IDO1 expression is a counter-regulatory mechanism to slow down potentially harmful over-activated immune responses. However, when the immune system attempts to fight a tumor, this counter-regulation is highly undesirable (151). In the majority of studies, an upregulation of IDO1 expression was associated with a poor clinical outcome (Table 1). Only in a small number of tumor entities, increased IDO1 activity was associated with a favorable prognosis (19, 54). The apparent inflammation-induced IDO1 expression in these patients probably indicates a stronger innate anti-tumor immune response.

It is suggested that IDO1 takes different positions in the three phases of cancer immunoediting: elimination, equilibrium, and escape (118). In the first phase (elimination), most tumor cells are recognized, and destroyed by the immune system. Low-level IDO1 production in the tumor microenvironment contributes to this tumor defense by inhibiting tumor proliferation (152). During the second phase (equilibrium), heterogeneity, and genetic instability progress in tumor cells that survived the elimination phase, thus enabling tumor cells to resist the immune response (153). In the last phase (escape), the tumor cells themselves as well as the tolerogenic immune cells produce large quantities of IDO1 (154), which results in immune tolerance described above (155, 156).

Due to these findings, inhibition of IDO1 as a therapeutic approach in cancer treatment has gained increasing attention in immuno-oncology. A recent study found that limitation of programmed cell death protein 1 (PD-1) inhibition might be due to an immunosuppressive tumor microenvironment based on IDO1 activation within macrophages (157). This suggests that IDO inhibition can be a potential therapeutic target in cancer patients, specifically in those who do not respond to immune checkpoint inhibitors. By now, clinical trials testing IDO1 inhibitors in combination with other chemotherapeutic or immunotherapeutic agents seem more promising than administration of IDO1 inhibitors alone. So far, five IDO1 inhibitors were studied as potential therapeutic options in cancer patients: indoximod [IDO pathway modulator; 1-methyl-D-tryptophan (1-MT)], epacadostat (selective IDO1 inhibitor; INCB024360), navoximod (GDC-0919), BMS-986205, and IDO1-targeting vaccines. All these IDO1 inhibitors were shown to be safe and well-tolerated (158–161). Epacadostat is the clinically most advanced IDO1 inhibitor and has been shown to inhibit tumor growth in mice models (162).

In human patients, epacadostat monotherapy was not effective (163, 164), while combined administration with PD-1 or cytotoxin T-lymphocyte-associated protein 4 (CTLA-4) inhibitors showed promising clinical activity in phase I/II studies (165–168). Unfortunately, a recent trial with combined administration of epacadostat with pembrolizumab found no superiority over pembrolizumab alone (169). Despite this setback, several ongoing trials investigate the effect of other (also structurally new) IDO1 inhibitors in combination with different immunotherapies (162).

## Indoleamine 2,3-dioxygenase 2, Tryptophan 2,3-Dioxygenase, and Kynurenine 3-Monooxygenase in Tumor Immune Tolerance

Until now, IDO2 has been investigated far less than IDO1. Although IDO2 is expressed by cancer cells, it does not contribute to the accumulation of Trp metabolites to the same extent as IDO1 (170, 171). However, it was recently implicated that IDO2 affects B cell-mediated autoimmunity (172), and also contributes to carcinogenesis in models of pancreatic cancers (173). Interestingly, IDO2-deficiency was predictive for disease-free survival in patients receiving adjuvant radiotherapy (173).

Recent studies revealed that TDO may also be involved in tumor immune-escape. It was demonstrated that TDO is expressed in various tumors including glial tumors (174), breast cancers (175), lung cancers (59), colorectal carcinomas (176), melanomas, bladder carcinomas, and hepatocellular carcinomas (177). In glial tumors, TDO activity suppressed the anti-tumor immune responses via increased Kyn production (174). TDO was shown to be a promising therapeutic target to improve immune response to cancer cells (178). A recent study by Schramme et al. demonstrated that TDO inhibition increases the antitumor efficacy of immune checkpoint inhibitors (179).

Also, KMO activity may be involved in tumor immune tolerance. Recent studies have shown that its overexpression is related to rapid cancer progression and a poor prognosis (49, 180). Similar to inflammatory-induced IDO1 expression, KMO expression is induced by inflammatory stimuli (181, 182). Interestingly enough, the non-steroidal anti-inflammatory drug diclofenac is capable of binding human KMO, thereby inhibiting its activity (183). Since there is evidence that diclofenac also exerts anti-cancer effects (184), a possible explanation might be its interaction with Trp metabolism. Diclofenac inhibits COX-2 related IDO1 expression and KMO expression, thus reducing the accumulation of Trp catabolites.

## Fatigue and Depression are Related to Immune Activation in Cancer Patients

Cancer related fatigue (CRF) is a complex multi-dimensional phenomenon that affects physical, cognitive and emotional activity, and behavior (185). It is associated with the cancer and its comorbidities themselves and often deteriorates during treatment (186). Actually, persisting fatigue limits the adherence of patients to cancer therapy (187). Chronic inflammation is proposed to be a leading cause of CRF. Higher inflammatory markers including IL-6, TNF-α, CRP, and neopterin were shown to correlate with fatigue in cancer patients prior to treatment, during treatment and also after treatment (62, 188–190).

Patients with lung cancer and moderate or severe fatigue are presented with lower Trp and hemoglobin concentrations, but with higher inflammatory markers (62). They furthermore assessed their QoL worse, and decreased QoL was associated with higher inflammatory markers and lower Trp concentrations. These results in 50 patients with lung cancer are well in line with earlier data showing significant correlations between fatigue/decreased QoL and immune-mediated Trp degradation in patients with different malignant diseases (85) as well as in patients with HIV-infection (191). Interestingly, correlations between inflammatory markers and decreased QoL were only seen in patients without antidepressant therapy in both HIV-infected and lung cancer patients. Also, in patients with colorectal cancer increased neopterin and decreased Trp levels correlated significantly with a decreased survival; QoL was worse in patients with low Trp (192).

A recent study in patients with solid tumors excluded patients with known depression or antidepressant treatment or established infection (90). Again, an association between immune activation and the QoL of patients as well as their depression susceptibility became evident. Fatigue was present in a high percentage of patients and was significantly associated with a decreased QoL, with decreased Trp and hemoglobin values (90). As low Trp or increased Kyn/Trp concentrations were associated with fatigue and decreased QoL, respectively, in several studies, this data indicates that immune activation and immune-mediated Trp degradation might contribute to the development of fatigue. Also, Kim and co-workers suggested a key role of inflammation-induced IDO-activation in CRF (193).

It is of importance that treatment with corticosteroids or anti-inflammatory drugs like celecoxib reduces fatigue in patients with advanced cancer (194, 195), suggesting that anti-inflammatory therapy improves fatigue by interfering with immune activation. A causal relationship between fatigue and immune activation has also been proposed in patients with other autoimmune diseases and infection (196) and treatment with TNF-α antagonists significantly reduces fatigue in patients with rheumatoid arthritis and psoriasis (197, 198).

Fatigue is one of the main symptoms of depression, which is another common comorbidity in subjects suffering from malignancies, affecting ~20% of the patients (99–101). Depression is probably not only due to emotional distress but also due to immunological mechanisms, which might negatively affect the QoL and increase all-cause mortality (199–201). Enhanced Trp breakdown as a consequence of immune activation has been proposed to play a crucial role in the development of depression in cancer patients (202–204).

Recently, correlations between inflammation markers (neopterin and CRP) and depression scores in a population of patients with solid tumors were reported, and particularly in male patients, lower Trp levels were associated with higher depression scores and stronger fatigue (90).

This clinical data fit well with results from animal experiments: Depressive-like behavior related to immune activation was demonstrated to be associated with an upregulation of IDO1 (205–207) as well as KMO (208–210). Peripheral administration of lipopolysaccharide activated IDO, resulting in a distinct depressive-like behavioral syndrome (205). Interestingly, IDO inhibition prevented the development of depressive-like behavior (211), while Kyn administration dose dependently induced depressive-like behavior. Also the anti-inflammatory cytokine IL-10 was able to normalize IDO1 expression, thus relieving depressive-like behavior in mice (212).

Depression is also related to enhanced Trp breakdown and immune activation in patients with HIV-infection (191, 213), as well as in patients receiving immunotherapy [e.g., IL-2 or INF-α; (77, 214)].

Immune activation probably affects the development of CRF and depression also by other mechanisms: Pro-inflammatory cytokines, for one thing, directly affect basal ganglia and dopamine function and, for another, activate sensory nerves. This results in production of pro-inflammatory cytokines and prostaglandins by microglia in the CNS, which then affect the functionality of neurons, thereby contributing to fatigue (215). Immune activation furthermore influences the biosynthesis of the catecholamines dopamine, epinephrine and norepinephrine and the neurotransmitter serotonin (216).

## Inflammatory-Induced Tryptophan Breakdown Contributes to the Development of Cancer Related Fatigue and Depression

There are several pathophysiological mechanisms, which might explain how Trp metabolites cause CRF or neurobehavioral symptoms related to CRF such as depression.

Trp is a crucial amino acid in brain homeostasis and a precursor for serotonin and melatonin synthesis. It can cross the blood-brain barrier; therefore, reduced Trp availability may contribute to serotonin dysregulation and neurobehavioral manifestations (217, 218). However, also the accumulation of downstream metabolites of the Kyn pathway is suggested to trigger neurobehavioral symptoms (204, 205).

QUIN, which is primarily produced by monocytes/macrophages and microglia, generates free radicals, causes structural changes, and is a selective agonist at the glutamate receptor sensitive to N-methyl-D-aspartate (NMDA receptor) (219). Its accumulation results in excitotoxicity, neuronal cell death and disturbs glutamatergic transmission (220). QUIN cannot cross the blood brain barrier, which is why only QUIN synthesized by microglia or monocytes/macrophages migrated to the CNS influences neuroimmunology (221). On the contrary, KYNA is considered as a neuroprotective Trp metabolite, because it acts as antagonist at the NMDA and other glutamate receptors (222). Previous studies have demonstrated that KYNA can protect against QUIN related neuronal damage (223). This balance between neurotoxic and neuroprotective effects is expressed by the QUIN/KYNA ratio and related to the grade of pathway activity, but also immune activation (224). It was shown that depressed patients have a higher QUIN/KYNA ratio compared to healthy controls, thus moving the balance toward the neurodegenerative effects (225). The imbalance of neurotoxic and neuroprotective Trp metabolites is suggested to play a major role in the development of neuropsychiatric symptoms including CRF and depression (226). 3-HK also exerts neurotoxic effects by causing lipid peroxidation (227).

Although immune system activation frequently coincides with fatigue or depression in cancer patients, it has to be kept in mind, that fatigue or depression also can develop isolatedly in patients with other predisposing conditions (like anxiety or little social support). Probably the development of neuropsychiatric disturbances and depression is alleviated in the presence of an activated immune system and accelerated Trp breakdown, but it must not necessarily lead to depressed mood. Maybe the handling of bad news is impaired if Trp and thus serotonin availability is low.

Additionally, also other factors, like psychosocial aspects including demographical factors (age, gender, culture/ethnicity and social support), behavior/well-being (composed of stress/distress—including spiritual, anxiety, sleep disturbance, coping style, and pain) but also functional status (performance status, physical activity level, physical functioning, and productivity/work) contribute to the development, severity, and duration of CRF and depression. Moreover, an imbalance in the autonomic nervous system, disturbances in the hypothalamic-pituitary-adrenal axis and circadian rhythm as well as hypoxia or anemia are key players in the pathophysiology of CRF and depression (228, 229). These factors might in fact enforce vicious circles, such as e.g., psychosocial stress triggers oxidative stress and inflammation, and thus tumor progression (201).

## Inhibition of Tryptophan Breakdown for Treatment of Fatigue and Depression

Experiments in mice demonstrated that the IDO pathway modulator indoximod inhibits depressive-like behavior (consecutive to bacterial infection) without altering the infectious immune response (211, 230). Moreover, the specific IDO1 inhibitor epacadostat was shown to reverse chronic social defeat in mice (231). Another interesting compound, which might target IDO, is the antibiotic minocycline, which was demonstrated to reduce IDO activation and thus prevent depressive-like behavior in animal studies (232–234). Minocycline was also able to decrease IDO expression and the formation of pro-inflammatory cytokines in LPS-treated monocytic human microglial cells (235–237), suggesting that IDO inhibition might be responsible for the anti-depressive effects of minocycline. Also, in humans a large and statistically significant antidepressant effect of minocycline has been observed when comparing to placebo [see review and meta-analysis by Rosenblat and McIntyre (238)]. Due to the good tolerability, future larger RCTs investigating the potential of minocycline (238), but also of other anti-inflammatory treatments (239) are considered. Contrary to these findings, a recent study with mice showed no improvement of cancer-related behavioral symptoms when inhibiting IDO1 (either by an unspecific or a specific IDO inhibitor). Mice treated with 1-MT even had slightly more treatment-associated burrowing deficits. Genetic deletion of IDO on the other hand had no effect on the behavior of mice, but was associated with a worse tumor outcome (240). In consideration of these conflicting data, more studies investigating effects of IDO inhibition in cancer are needed. Clinical trials targeting TDO revealed antidepressant effects as well as amelioration of neurodegeneration following TDO inhibition, and seem to be a promising therapeutic target in cancer patients, especially with neurobehavioral symptoms (241, 242).

Inhibition of KMO also seems to be a possible therapeutic approach in the treatment of fatigue and depression by shifting Kyn metabolism toward the enhanced production of neuroprotective KYNA while decreasing production of neurotoxic QUIN. A recent mice trial revealed that KMO gene deletion substantially reduces 3-HK and QUIN concentrations while elevating KYNA concentrations (243). It was further shown to ameliorate neurodegeneration in patients with Alzheimer's and Parkinson's diseases (242). Therefore, KMO inhibition may be a promising therapeutic target in inflammation-related fatigue or depression by reducing generation of the neurotoxic Trp metabolites 3-HK and QUIN.

Another recent study showed decreased IDO1 and KMO expression in the murine brain as well as decreased IDO1 and IDO2 expression in human peripheral blood mononuclear cells as a consequence of antidepressant treatment (244, 245). This, in turn, demonstrates that reduction of psychosocial stress can also reduce inflammation-related factors.

## Nutrition, Microbiome, and Physical Activity and its Association With Tryptophan Breakdown, Fatigue, and Depression

Monoaminergic antidepressants and also omega-3 fatty acids were demonstrated to reduce neurotoxic effects related to Trp breakdown (246). Omega-3 fatty acids contribute to the beneficial effects of the Mediterranean diet, which is regarded as anti-inflammatory diet (247). High adherence to this diet is linked to a lower risk of developing cancer and to a reduced cancer mortality in observational studies (248). A “Western” diet rich in refined sugars and long chain fatty acids and with low fiber content on the other hand enforces a type 1 pro-inflammatory state (249). Mouse experiments furthermore showed that Western diet exposure exacerbated hippocampal and hypothalamic proinflammatory cytokine expression and brain IDO activation after immune stimulation with LPS (250). Inflammation-induced Trp degradation in humans might then further intensify subdued psychosocial factors such as mood, negative thoughts and lack of energy or simply make patients more susceptible to them.

In fact, diet and the gut microbiome may influence inflammation and Trp metabolism by several ways (251): Microbiota metabolize phytochemicals (e.g., in vegetables) to indoles, which activate AhR as ligands, while other microbial-derived metabolites such as the short chain fatty acids butyrate, propionate, and acetate importantly mediate the crosstalk between host-microbiota and thereby have immune modulating effects (251). Actually Trp metabolic pathways are regarded as key biochemical pathways influencing the microbiota-neural-immune axis by translating information on the nutritional, inflammatory, microbial, and emotional state of the organism to the immune system (252–254) and by modulating intestinal immune response (251).

A recent review by Weber et al. proposed that preclinical and several clinical studies argued for the use of a ketogenic diet (KD) in combination with standard therapies in patients with cancer (255): KD had the potential to enhance the antitumor effects of classic chemo- and radiotherapy and to increase the QoL of patients (255). However, the heterogeneity between studies investigating these effects and low adherence to diet limit the current evidence (256). Interestingly, KD was shown to positively influence the Kyn pathway in rats (257). Increased β-hydroxybutyrate concentrations and an increased production of the neuroprotective KYNA were found in rat brain structures as a consequence of KD (258, 259). Also, a recent study in children revealed that Kyn levels significantly decreased and KYNA levels significantly increased 3 months after starting a KD (260).

Significant differences regarding Trp metabolism were reported between a low-glycemic load dietary pattern (characterized by whole grains, legumes, fruits, and vegetables) and a diet high in refined grains and added sugars on inflammation and energy metabolism pathways (261). In line with results of this study, a Mediterranean diet and other plant-based diets have been proposed to reduce fatigue in cancer survivors (262).

As cancer cells are very vulnerable to nutrient deprivation (especially glucose), fasting or fasting-mimicking diets (FMDs) might be another effective strategy to generate environments that can reduce the capability of cancer cells to adapt and survive and thus improve the effects of cancer therapies (263). Further studies investigating the effects of FMDs on Trp catabolism in the tumor microenvironment might therefore provide interesting new insights for future treatment approaches.

Besides, treatment with probiotics might be beneficial for cancer patients: In colorectal cancer survivors, probiotics (*Lactobacillus acidophilus* and *rhamnosus*) improved CRF, irritable bowel syndromes and QoL significantly in a double-blind placebo-controlled study (264); furthermore, probiotics and also melatonin supplementation appear to alleviate side effects of radiation therapy (265). Probiotic supplementation with *Lactobacillus plantarum* in combination with SSRI treatment improved cognitive performance and decreased Kyn concentrations in patients with major depression [compared to SSRI treatment alone, (266)]. Supplementation with a multispecies probiotic had a beneficial effect on Trp metabolism in trained athletes (267) and influenced Trp degradation and gut bacteria composition in patients with Alzheimer's disease (268). Additionally, highly adaptive lactobacilli where shown to produce the AhR ligand indole-3-aldehyde, which enabled IL-22 transcription for the fine tuning of host mucosal reactivity (269). Conclusively, these studies indicate that beneficial effects of probiotics on fatigue or depression might be due to alterations of Trp metabolism or anti-inflammatory effects [see review by (270)]. However, evidence is limited due to the heterogeneity of clinical trials. Therefore, further well-designed longitudinal placebo-controlled studies are desperately needed (271, 272).

Also, a recent review of clinical trials that assessed nutritional interventions for preventing and treating CRF suggests that supplementation with probiotics but also ginseng, or ginger may improve cancer survivors' energy levels and that nutritional interventions, alone or in combination with other interventions should be considered as therapy for fatigue in cancer survivors. Nevertheless, there is lacking evidence to determine the optimal diet to improve CRF in cancer patients (262, 273). Furthermore, also physical activity, psychosocial, mind-body, and pharmacological treatments have been proven to be effective (187).

Physical exercise also affects Trp metabolism and thereby might improve fatigue and depression. As this subject has been discussed elsewhere recently (274, 275), it will be discussed only briefly hereafter. Physical activity increases Trp availability in the brain, which results in an increased 5-HT synthesis and anti-depressant effects (276). Increased muscle use of branched-chain amino acids (BCAAs) favors the passing of Trp through the blood-brain barrier (277). In addition, endurance exercise increases concentrations of circulating free fatty acids, which displaces Trp from albumin, thus increasing free Trp concentrations (278). Additionally, physical activity increases the expression of kynurenine aminotransferases, which enhance the conversion of Kyn into KYNA (unable to cross the blood-brain barrier), thus protecting the brain from stress-induced changes (279). Interestingly, intense physical exercise induces the formation of several pro-inflammatory cytokines (280), which in turn activate IDO1 and Trp breakdown.

## Immune Activation Causing Tryptophan Degradation and (Consequently) Anemia

Another common comorbidity in cancer causing fatigue is anemia (95, 281). Anemic cancer patients have a worse QoL, an adverse outcome as well as a reduced rate of local tumor control compared to non-anemic cancer patients (282, 283).

Anemia is the most common “hematological complication,” found in ~40–64% of patients with malignant diseases (94) and is mostly due to anemia of chronic disease (ACD) (284). ACD is caused by enhanced formation of pro-inflammatory cytokines, which can on the one hand directly inhibit erythropoiesis and on the other hand restrict the availability of iron for erythropoiesis. The latter is caused by an increased uptake and retention of iron within the cells of the reticuloendothelial system together with a suppression of iron absorption in the duodenum. The master regulator of iron homeostasis, hepcidin, has a decisive role in these processes. Similarly to Trp breakdown, this is initially a protective mechanism of the immune system to restrict available iron from microbes or tumor cells (285, 286).

IFN-γ, one of the main cytokines of Th1 type immune response, activates IDO and neopterin formation in hematopoietic stem cells and also exerts an influence on the proliferation of various stem cell populations (287). The intravenous injection of neopterin into mice resulted in a prolonged decrease in the number of erythroid progenitor cells and increased the number of myeloid progenitor cells (CFU-GMs) by activating stromal cells (288).

Trp metabolites like Kyn, on the other hand, increase hepcidin expression and inhibit erythropoietin (EPO) production by activating AhR (289). AhR competes with hypoxia-inducible factor 2α (HIF-2α), the key regulator of EPO production, for binding with HIF-1β (289, 290). Well in line with this finding, Kyn/Trp and neopterin were shown earlier to be associated inversely with hemoglobin concentrations and positively with hepcidin concentrations in patients with HIV-infection before antiretroviral therapy (287). Antiretroviral treatment slowed down immune-mediated Trp catabolism and improved iron metabolism and anemia (287).

Interestingly, in patients with different malignant diseases, increased Kyn/Trp and neopterin concentrations also coincided with lower hemoglobin values (85). Also, recent data confirms that anemic cancer patients present with higher inflammatory markers and a higher Kyn/Trp than non-anemic individuals (90). The same is also true for patients with anemia due to inflammation (291) and for HIV-infected patients (191).

Also, QUIN was shown to inhibit EPO production (292) by stimulating the production of nitric oxide (NO) (293) and inducing HIF-1α degradation (294).

In patients with myelodysplastic syndromes, a fundamental role for Trp metabolized along the serotonin pathway in normal erythropoiesis and in the physiopathology of MDS-related anemia was demonstrated recently: Decreased blood serotonin levels were related with impaired erythroid proliferating capacities, and treatment with fluoxetine, a common antidepressant, was effective in increasing serotonin levels and the number of erythroid progenitors (295).

Low serotonin concentrations are also associated with the development of depression. Vulser et al. actually showed a considerable association between anemia and depression in otherwise healthy adults (296). Increased Trp degradation might therefore be a connection between anemia and depression.

These findings show that impaired Trp availability but also accumulation of Trp metabolites, may affect erythropoiesis. In cancer patients, tumor cells produce TDO and IDO1, and both are equally capable of producing Kyn (174). However, they may only contribute to local Trp degradation and do not influence systemic Trp breakdown. On the other hand, IDO1 activity is also stimulated by the activated immune system and thereby contributes to systemic Trp catabolism. Therefore, inflammation-induced IDO1 activation and consecutive Trp breakdown might influence erythropoiesis. The most common symptom of anemia is fatigue, which is why both ACD and inflammation-induced Trp breakdown may be major contributors to overall-fatigue in patients with malignant diseases.

## Conclusion

Inflammation-induced Trp breakdown in cancer patients is considered to play a key role in the pathophysiology of tumor immune tolerance. Accumulation of Trp metabolites as well as impaired Trp availability suppress the tumor immune response and may also greatly contribute to the development of comorbidities such as fatigue, depression, or anemia, which are all common in patients with malignancies. Although anemia is primarily caused by the enhanced immune response itself, inflammatory-induced Trp degradation may also be involved strongly. Studies have shown that inhibition of Trp breakdown might be a promising therapeutic option in cancer patients to counteract the immunosuppressive tumor microenvironment. Especially cancer patients with no response to immune checkpoint inhibitors might benefit from an additional IDO1 inhibition. Moreover, there is evidence that inhibition of IDO1, TDO, and KMO or other interventions targeting Trp metabolism (like diet or probiotics) may further improve neurobehavioral manifestations including CRF or depression. Further studies investigating the effects of IDO1, TDO, or KMO inhibition on tumor immune response should also take the impact on neurobehavioral manifestations into consideration.

## Author Contributions

LL and KK wrote the manuscript. PK, EE, WW, DF, and GW critically read and revised the paper. All authors listed approved the submitted version for publication.

### Conflict of Interest

The authors declare that the research was conducted in the absence of any commercial or financial relationships that could be construed as a potential conflict of interest.
